# Animal study assessing safety of an acoustic coupling fluid that holds the potential to avoid surgically induced artifacts in 3D ultrasound guided operations

**DOI:** 10.1186/1471-2342-14-11

**Published:** 2014-03-25

**Authors:** Asgeir S Jakola, Arve Jørgensen, Tormod Selbekk, Ralf-Peter Michler, Ole Solheim, Sverre H Torp, Lisa M Sagberg, Petter Aadahl, Geirmund Unsgård

**Affiliations:** 1 Department of Neurosurgery, St.Olavs University Hospital, Trondheim, N-7006, Norway; 2 MI Lab, Norwegian University of Science and Technology, Trondheim, Norway; 3 Department of Neuroscience, Norwegian University of Science and Technology, Trondheim, Norway; 4 National Competence Centre for Ultrasound and Image-guided Therapy, Trondheim, Norway; 5 Department of Diagnostic Imaging, St.Olavs University Hospital, Trondheim, Norway; 6 Department of Medical Technology, SINTEF, Trondheim, Norway; 7 Department of Neurology and Clinical Neurophysiology, St.Olavs University Hospital, Trondheim, Norway; 8 Department of Laboratory Medicine, Children’s and Women’s Health, Norwegian University of Science and Technology, Trondheim, Norway; 9 Department of Pathology and Medical Genetics, St.Olavs University Hospital, Trondheim, Norway

**Keywords:** Brain imaging, Brain tumor, Intraoperative imaging, Ultrasound

## Abstract

**Background:**

Use of ultrasound in brain tumor surgery is common. The difference in attenuation between brain and isotonic saline may cause artifacts that degrade the ultrasound images, potentially affecting resection grades and safety. Our research group has developed an acoustic coupling fluid that attenuates ultrasound energy like the normal brain. We aimed to test in animals if the newly developed acoustic coupling fluid may have harmful effects.

**Methods:**

Eight rats were included for intraparenchymal injection into the brain, and if no adverse reactions were detected, 6 pigs were to be included with injection of the coupling fluid into the subarachnoid space. Animal behavior, EEG registrations, histopathology and immunohistochemistry were used in assessment.

**Results:**

In total, 14 animals were included, 8 rats and 6 pigs. We did not detect any clinical adverse effects, seizure activity on EEG or histopathological signs of tissue damage.

**Conclusion:**

The novel acoustic coupling fluid intended for brain tumor surgery appears safe in rats and pigs under the tested circumstances.

## Background

**Figure 1:**
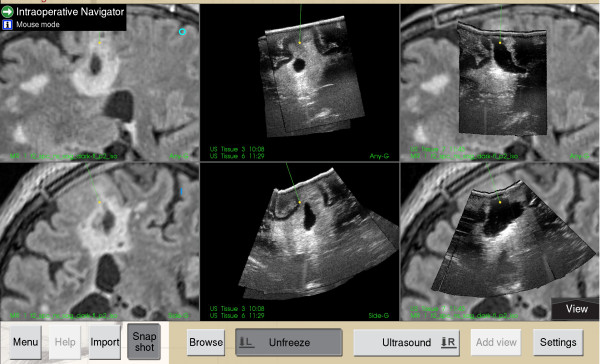
**Ultrasound imaging during glioma resection.** Screen dump from the ultrasound based neuronavigation system (SonoWand®). Images to the left are preoperative MRI demonstrating a hyperintense region suspect of diffuse glioma (above; sagittal view, below; coronal view). Images in the middle represent the corresponding ultrasound images taken intraoperatively before resection. Pictures to the right demonstrate corresponding ultrasound images approximately midway in the resection. Notice the bright-rim in the bottom of the cavity, representing an artifact, making the interpretation of hyperintense areas that could represent tumor on the images somewhat more difficult.

For glial brain tumors the extent of resection is associated with survival [1,2]. However, extensive resection should not jeopardize function [3,4]. Tools for enhancing surgical resection of brain tumors, in particular gliomas, are increasing [4-7]. Ultrasound is currently used as a tool for providing 2D or 3D images for the purpose of tumor localization and resection control. Ultrasound has the potential to guide resections and increase resection grades, and thereby increase survival [8]. While ultrasound image quality is linked to resection grades in glioma surgery the image quality may sometimes be threatened by artifacts [9], most commonly seen towards the end of an operation, reducing the specificity [10,11]. When ultrasound is used for resection control the resection cavity is filled with saline to provide acoustic coupling between the ultrasound transducer and tissue. However, attenuation of acoustic waves is very low in saline compared to the brain and this difference in attenuation may cause artifacts that degrade the ultrasound images. Such artifacts are seen as high-intensity signals at the resection cavity wall and beyond, potentially masking a small tumor remnant, or even mimic tumor when there is none, and generally make the image interpretation more difficult (Figure 1).

This research group has developed a patent pending fluid intended for use in the resection cavity instead of saline. The effect on image quality has been tested in laboratory measurements using phantoms and fresh animal cadavers. The fluid seems to enhance ultrasound image quality by the reduction of ultrasound artifacts around the resection cavity. We believe that the acoustic fluid will make image interpretation easier towards the end of surgery by easing delineation of normal brain tissue and tumor tissue, thus improving resection grades and improving safety.

In this two-step animal safety study we aimed to examine if the newly developed acoustic coupling fluid has any harmful effect related to its intended use.

## Materials and methods

### Study design

We designed a two-phased animal study with assessment in eight rats first, and if no adverse reactions were detected, pigs were to be included. Animals were successively included and no animals were excluded after the initiation of the experiments. The rat experiment was a non-randomized experimental study with each rat being its own control as the acoustic fluid was injected in one hemisphere and isotonic saline in the other. The pig experiment included six animals in a non-randomized consecutive study with no controls.

### Experimental acoustic fluid

The (developed) acoustic coupling fluid is based on substances available in the pharmacy for intended use by humans. In the developed fluid we tested a pharmaceutical grade triglyceride and a pharmaceutically acceptable emulsifier and a pharmaceutically acceptable humectant. The main ingredient of the fluid we use is purified soybean oil in addition to lecithin and glycerol. These “active ingredients” is then diluted with a crystalloid solution (e.g. physiologic saline or Ringer’s lactate), the more you dilute the lower the attenuation will be. From attenuation measurements in the laboratory and ultrasound imaging on fresh animal cadavers we established a certain blend of the above mentioned ingredients that should attenuate sound waves similarly as the human brain. See also Selbekk et al. [12].

### Experimental animals

In the first phase of the study, acoustic fluid was injected in the frontal cortex of one randomly chosen hemisphere and control fluid in the contralateral hemisphere of eight adult female Sprague-Dawley rats (Taconic, Denmark). In the second phase, acoustic fluid was injected in the cisterna magna of six female domestic farm pigs, *Sus scrofa domesticus* (Nortura, Norway). The acclimatization length was four days for three pigs and five days for the other three pigs. One additional pig served as control for the model of assessing epileptogenicity by injecting penicillin G instead of the acoustic fluid into the cerebrospinal fluid in cisterna magna. Rats were housed in groups of three per cage in an animal facility and pigs were housed together except for the first day after the intervention when they were kept separated. Illumination was controlled on a 12:12-h light-dark cycle at room temperature 21°C and humidity 60% ± 2 SD. Animals had free access to water and a pellet rodent or pig diet.

### Injection of fluid in brain parenchyma of rats

The rats were anesthetized with a gas mixture of 5% isoflurane (Abbot Scandinavia, Solna, Sweden), O_2_ 0.25 L · min^-1^ and N_2_O 0.55 L · min^-1^. After induction of anesthesia all rats received 0.012 mg atropine subcutaneous injection diluted in 1 mL NaCl 0.9% solution. The isoflurane concentration was adjusted down gradually to 1.8%, not faster than 0.5% each minute. The rat’s head was fixed in horizontal position by a stereotactic frame. The procedure was done under sterile conditions, and an operating microscope was used during the surgical intervention. After exposing of the cranial vault, xylocain 10% was sprayed onto the surface and two 1 mm wide holes were drilled. Dura mater was carefully removed using a dura hook. The fluids were injected into the frontal lobe cortex of both hemispheres at 0.16 μL · min^-1^ by means of a 33 gauge internal cannula (C315I; Plastics One) inside a 26 gauge outer cannula (C315G; Plastics One) with the tip of the inner cannula protruding 0.9 mm out of the outer cannula. The cannula was connected by polyethylene tubing to a 25 μL Hamilton syringe in a CMA/100 microinfusion pump (Carnegie Medicine, Stockholm, Sweden). The entry point was 1.5 mm posterior to Bregma, 3.4 mm to both sides of the sagital suture, and 1.5 mm deep into the somatosensory cortex. A total volume of 1.00 μL fluid (either saline or acoustic fluid) was injected, and the cannula was retracted five minutes after each injection to prevent backflow of the fluid. The skin was sutured before the rats returned to their cages. Core temperature was maintained during the procedure using a heating pad and monitored using a rectally inserted thermometer. The equipment and procedure for injection were the same as used by Hafting et al. [13].

### Subarachnoid injection of fluid in cisterna magna of pigs

The pigs were sedated with a combination of 10 mL azaperone (40 mg · mL^-1^) and 2 mL diazepam (5 mg · mL^-1^). Intravenous access was established via an ear vein. Just before intubation 1 mL atropin (1 mg · mL^-1^), 5 mL ketamin (50 mg · mL^-1^), 5 mL fentanyl (50 μg · mL^-1^) and 5 mL thiopentothal (25 mg · mL^-1^) were administered. After intubation the pigs remained anesthetized with 1–3% isoflurane (Abbot Scandinavia, Solna, Sweden), adjusted individually, 50% oxygen with the remaining being medical air 3 L · min^-1^ (AGA AS, Linde Healthcare, Norway). Pigs were positioned in prone position on a heating blanket with the neck flexed over the operating table to facilitate puncture of cisterna magna. Fluoroscopy was utilized for guidance in all animals. After verification of needle position in cisterna magna a total amount of 2 mL cerebrospinal fluid was collected. This equals 10% of the total CSF volume in pigs which is approximately 20 mL [14]. With the needle in place, 1 mL of the acoustic fluid was slowly injected and flushed with 1 mL of autologous cerebrospinal fluid. After the injection all animals were observed for 20 minutes while monitoring oxygen saturation and heart rate with 3-lead ECG continuously. After the observation period isoflurane was stopped. The pigs were extubated when they were capable of unassisted breathing and protection of airway.

### Electroencephalography recordings in pigs

**Figure 2:**
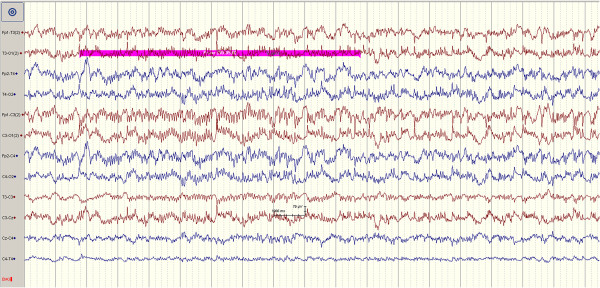
EEG registrations immediate after the first injection of penicillin G leading to generalized rhythmic fast sharp activity.

**Figure 3:**
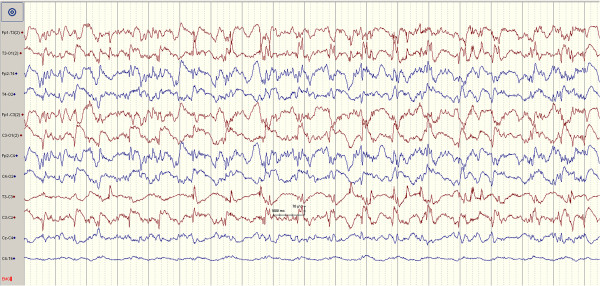
EEG registrations after the second injection of penicillin G leading to seizure activity.

Scalp electroencephalography (EEG) was recorded throughout the procedure in all pigs. The EEG protocol we utilized equals the EEG protocol in use for children at St.Olavs Hospital. A Schwarzer ED 21 (Picker International GmbH) was used to record 12 channel EEG (longitudinal bipolar, 10-20 electrode system, two common central scalp reference electrodes were applied, the filter was 0.5–70 Hz, sensitivity 100 mV · cm^-1^) which was digitized (Rhythm version 9, Stellate Systems) using a sampling rate equal to 200 · s^-1^ for eight minutes. Electrode placements were: Fp1-T3, T3-O1, Fp2-T4, T4-O2, Fp1-C3, C3-O1, Fp2-C4, C4-O2, T3-C3, C3-Cz, Cz-C4 and C4-T4. Subcutaneously needle electrodes were used. EEG was recorded throughout the injection procedure in all pigs. One pig served as control with the purpose to validate the model. In this pig 1 mL penicillin G (1 000 000 U · mL^-1^, diluted in 0.9% NaCl) was injected at two time points with 20 minutes interval. After the first injection the pig developed tachycardia and generalized rhythmic fast sharp activity (Figure 2). After the second injection there was after three minutes markedly pathological EEG (generalized spikes, spike/wave and slow waves) consistent with ongoing seizure activity (Figure 3). After additional seven minutes the pig developed frequent widespread and small muscle contractions, and the pig was then euthanized according to protocol with an overdose of 10% pentobarbital.

### Follow-up and assessment of animals

In rats, weight was measured prior to, right after, and nine days after the procedure. All animals, rats and pigs, had supervision at least two times daily and any abnormal behavior and signs of CNS affection after the procedure was monitored and registered by a veterinarian. In rats, general behavior, the level of stress and activity and animal appearance like body posture and fur (bristling or not) were registered. Behavior in pigs was systematically scored twice daily to enable capturing of any abnormal behavior or negative trends (Table 1).

**Table 1:** Scores of symptoms in pigs post-procedure

	**Day 2**	**Day 4**	**Day 6**	**Day 8**	**Day 10**	**Day 12**
**Pig 1**	0/12	0/12	1/12	1/12	1/12	1/12
**Pig 2**	0/12	1/12	0/12	0/12	0/12	0/12
**Pig 3**	0/12	0/12	0/12	0/12	0/12	0/12
**Pig 4**	1/12	2/12	0/12	0/12	0/12	0/12
**Pig 5**	0/12	0/12	0/12	0/12	0/12	0/12
**Pig 6**	2/12	2/12	1/12	0/12	0/12	0/12

The score was predefined by a veterinarian and the score was based on: appearance, behavior, nutritional status and relevant clinical signs (gait disturbances and/or seizure). For each dimension a range from 0 (normal) to 3 (markedly abnormal) was possible giving a range from 0-12 on each observation. The scores presented were from the assessment where the individual pig reached the highest sum (most symptoms). A total score of 0-3 was predefined as normal unless the pig scored 3 in one dimension (severe symptoms). A total score higher than 3 or maximal score in one dimension warranted a consult with a veterinarian.

### Brain biopsy of rats

The whole brain was acquired the ninth day after the surgical procedure. Isoflurane (Abbot Scandinavia, Solna, Sweden) was used to induce anesthesia, followed by intraperitoneal injection of an overdose of 10% pentobarbital (300 mg · kg^-1^). When the rats stopped breathing, the thorax was immediately opened and 100 mL phosphate-buffered saline was injected through the left cardiac ventricle while the heart was still beating, followed by 100 mL injection of 4% formalin for intravascular fixation of the brain parenchyma, a method adapted from Wideroe et al. [15]. Injection rate was set to 35 mL · min^-1^. An incision in the right atrium allowed solution to leave the circulation, thus avoiding volume overload and subsequent tissue damage. The head was then decapitated and embedded in 4% buffered formalin for two weeks prior to histopathological analysis.

### Brain biopsy of pigs

The brain biopsies were acquired at day 13 for three pigs and at day 16 for the other three. The pigs were sedated with 2 mL stesolid (5 mg · mL^-1^), 4 mL azaperone (40 mg · mL^-1^), 14 mL ketamin (50 mg · mL^-1^) and later euthanized with 40 mL 10% pentobarbital. Shortly after injection a large craniectomy was performed. Sampling of dura and brain parenchyma both above and below the tentorium was performed and acquired biopsies were embedded in 4% buffered formalin. In addition, plexus choroideus were sampled in all pigs.

### Assessment of histopathology

In the rat experiment the brain was cut in coronal sections (2 mm) and embedded in paraffin. From the paraffin-blocks 4 μm thick sections were cut, stained with haematoxylin-eosin, and examined microscopically. To assess processes such as gliosis, fibrosis or inflammation immunohistochemistry was performed using antibodies against lymphocytes (CD3 for T-cells and CD20 for B-cells), microglia/macrophages (CD68), and reactive astrocytes (glial fibrillary acidic protein (GFAP)).

Tissue samples of pig brain from supratentorial, infratentorial and brain stem region were fixed in buffered 4% formalin and embedded in paraffin. Paraffin-sections of 4 μm thickness were cut, stained with haematoxylin-eosin for microscopical analyses. The sections were also incubated with antibodies against GFAP, CD45 (leukocyte common antigen), and CD68 (microglial activation). The immunostaining was carried out on a DAKO Autostainer (Dako, Glostrup, Denmark). Visualization of immunoreactivity was performed with DAKO EnVision system with diaminobenzidin as chromogene. Sections were counterstained with haematoxylin. Positive controls were included in each staining run. In the negative controls the primary antibodies were omitted.

The neuropathologist (SHT) was blinded with respect to all data concerning the animal status and which side it was saline or acoustic fluid injection in the rat experiment.

### Statistical considerations

As the primary end-points were qualitative in nature (animal behavior, EEG registrations, histopathology and immunohistochemistry) we only present descriptive analysis. Average is presented as mean ± standard deviation.

### Ethical approval

The experimental protocols were reviewed and approved by the Norwegian Committee for Animal Experiments, and conform to the European Convention for the Protection of Vertebrate Animals Used for Experimental and other Scientific Purposes.

## Results

### General

The mean weight of the eight rats was 273 g ± 8 before the procedure and 267 g ± 8 after nine days post-procedure. The mean weight of the six pigs was 37 kg ± 2 before the procedure and 43 kg ± 4 at end of follow-up.

### Animal behavior

In rats, no apparent discomfort or altered behavior was registered during the follow up assessment. In pigs the post-procedure behavior was scored systematically (Table 1) without detection of adverse events (score ≥ 3/12).

### EEG registrations

**Figure 4:**
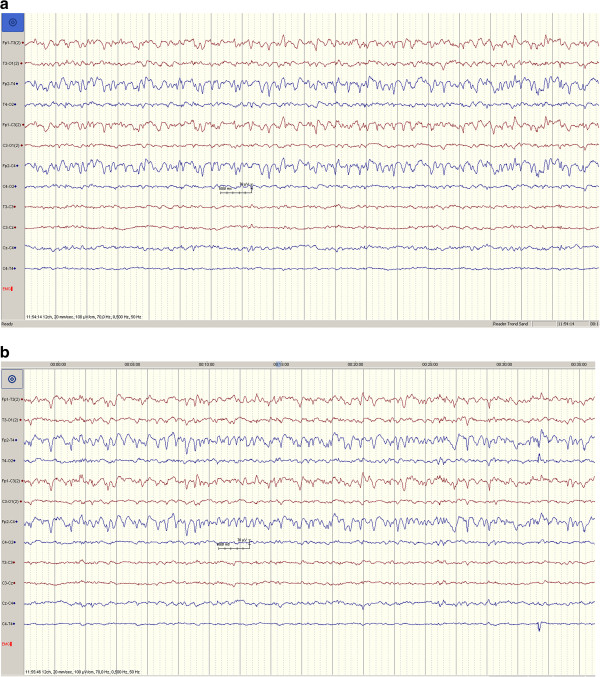
**EEG registrations in pig 3. a)** Before the procedure. Synchronous background activity 4-6 Hz with highest amplitudes in the frontotemporal region. **b)** After injection of the acoustic coupling fluid. There was no evidence of seizure activity on EEG.

In the six animals receiving injection of the acoustic fluid we have five EEG registrations with good quality. Median registration time was 43 minutes (range 26 – 54 minutes) with all pigs having more than 20 minutes of registration post-injection. For unknown reasons one EEG recording disappeared at attempt of storing. This pig (pig 5) did not appear any different with respect to heart rate, oxygen saturation or behavior after the procedure. The pig had an uneventful immediate recovery. In the other five pigs there were EEG recordings of good quality and there were no signs of seizure activity. Figure 4 demonstrates the pre- and post-procedure EEG in pig 3.

### Histopathological assessment

**Figure 5:**
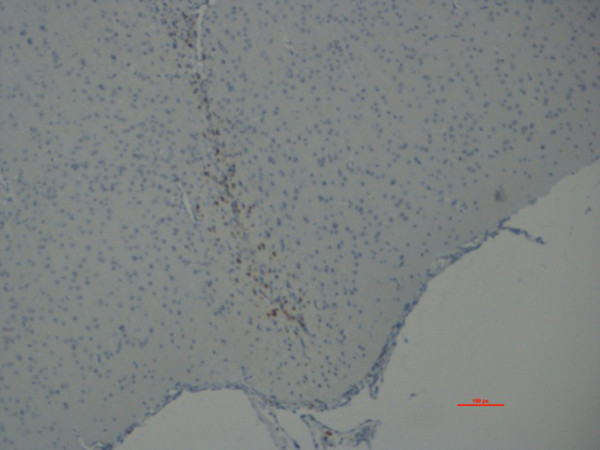
**Histology of injection site of the acoustic fluid in rat.** There was a minor macrophage response just around the needle site which is a normal reaction to brain trauma. Except from this there was no evidence of any reactions around the needle site.

No inflammation or tissue damage was seen after injection of the acoustic or control fluid in rat brain parenchyma with any of the techniques used. Figure 5 demonstrates the area of injection without any reactions. In pigs microscopical analyses did not reveal any kind of inflammation, neither in meninges, nor in brain tissue or vessels. Furthermore, no gliosis or microglial response was observed after immunohistochemistry. In the brain-stem biopsy in pig 4 a small focal area was observed with a few lymphocytes, eosinophiles, macrophages and slight spongiforme changes (subtle loss of neural tissue). No neutrophile granulocytes were observed. This focal area appeared microscopically to be of a chronic character and not due to the procedure.

## Discussion

In this safety study in rats and pigs we were unable to detect adverse clinical events of the newly developed acoustic coupling fluid for improving image quality in ultrasound-guided operations. Intra-procedure EEG was also unable to demonstrate any signs of epileptogenic effects. Also, there were no signs of acute inflammation in histopathological samples.

The intention with this testing was a relevant and sophisticated administration of the acoustic fluid (intraparenchymal and subarachnoidal) in two different species. Thus, we examined a local effect in rats and a more regional effect in pigs. The administration methods were chosen to minimize procedural damage, thereby enhancing post-procedural observation. The refined assessment was important to be able to detect any subtle damage to nervous tissue or its surroundings. Also, such methods are also in line with the 3R’s (refinement, reduction, replacement) in animal research.

Although no adverse events or signs of toxicity were observed in the two animal models, toxicity may be both dose-dependent and time-dependent. In rats 1 μL was injected intraparenchymal (approximately 1/2000 of the mean brain volume) and in pigs 1 mL was administered into the subarachnoid space (approximately 5% of total CSF volume) [14]. This would equal accumulation of 7.5 mL in the human CSF. Since the acoustic coupling fluid is likely to be opaque, a prerequisite for the usefulness would be 3D ultrasound imaging so that the region of interest in the ultrasound image can be retrieved. With ultrasound it is possible to update the image information several times during a brain tumor resection to follow the progression of tumor resection and to correct for brain shift [6]. Thus, repeated administration and removal of the acoustic fluid will be carried out. Acquisition of a 3D ultrasound image recording typically takes 2-3 minutes. Thereafter the coupling fluid will be removed with suction and irrigation. Topical accumulation of the fluid is unlikely since the surgeons after image acquisition would want to remove the fluid since it is opaque. Also, most of the fluid will presumably stay in the resection cavity since a horizontal craniotomy is most often attempted when brain tumor resections are facilitated by ultrasound [6]. The experience in animals is that the fluid is easily removed by suction and flushing with Ringer’s solution. A potential pitfall for excessive accumulation of the fluid to levels higher than tested in the animals could be in tumors where the ventricles are opened as a result of tumor resection. This could be the case in tumors similar to the situation seen in Figure 1. In these situations special care is necessary and until further safety data is available our recommendation is that fluid should not be administered *after* opening the ventricles.

The strengths of the model are testing of both rats and pigs in addition to use of relevant application methods (intraparenchymal and subarachnoid space) with sophisticated methods and the use of EEG monitoring. However, the drugs used to sedate the pigs could possibly increase the seizure threshold and mask EEG disturbances. Also, due to the number of tested animals it remains a possibility that more rare events may have gone unnoticed. However, further animal testing for rare events or reactions due to very high doses or delayed effects was considered less relevant because of the benign profile seen in our experiments. Still, translating positive therapeutic findings or negative toxicity findings from animal to humans are naturally always subject to uncertainty due to the inherent biological differences. We decided that the potential gain of further animal testing was limited and consequently we designed a study assessing safety in selected brain tumor patients. Recently this protocol was approved by the Norwegian Medicines Agency and the local ethical committee and we are now in the beginning of a phase 1 safety study (EudraCT number 2012-005567-27).

The acoustic fluid holds potential to enhance image quality when using 3D ultrasound in brain tumor resections. We hope this will make image interpretation easier at the end of a brain tumor resection, an improvement that could be of particular importance for users with limited experience in using ultrasound guided surgery. Improved imaging near the end of the surgery could be associated with improved resection grades that again improves clinical outcome [2,4,5,16]. We now hope to translate these positive findings from simulation and the animal models to highly selected patients with suspected high-grade glioma to further evaluate the potential benefits while carefully monitor any potential adverse events.

## Conclusion

We have in rats and pigs demonstrated that the newly developed acoustic coupling fluid intended for use in ultrasound-guided brain tumor surgery appears safe under the tested circumstances. We are now in the beginning of a phase 1 clinical study where different concentrations of the acoustic coupling fluid is tested in selected patients at our institution in order to assess safety and impact on ultrasound image quality.

## Competing interests

Geirmund Unsgård and Tormod Selbekk have submitted a patent application with respect to the novel acoustic fluid.

## Authors’ contributions

ASJ: planned the experiments, carried out the animal experiments, collected data, drafted the article, submitted manuscript on behalf of authors. AJ: planned the experiments, carried out the rat experiments, revised the manuscript. TS: planned the experiments, responsible for the development of the acoustic fluid, assisted in the experiments, revised the article. RPM: assisted in the experiments, revised the article. OS: planned the experiments, revised the article. SHT: did the histopathological analysis, revised the article. LMS: planned and assisted in the experiments, revised the article. PA: planned and assisted in the experiments, revised the article. GU: planned experiments, responsible for the development of acoustic fluid, revised the article. All authors read and approved the final manuscript.

## Pre-publication history

The pre-publication history for this paper can be accessed here:

http://www.biomedcentral.com/1471-2342/14/11/prepub
